# Equity of access to reproductive health services among youths in resource-limited suburban communities of Mandalay City, Myanmar

**DOI:** 10.1186/1472-6963-12-458

**Published:** 2012-12-15

**Authors:** Phyu Phyu Thin Zaw, Tippawan Liabsuetrakul, Thien Thien Htay, Edward McNeil

**Affiliations:** 1 Department of Medical Research (Upper Myanmar), Pyin Oo Lwin, Myanmar; 2 Epidemiology Unit, Faculty of Medicine, Prince of Songkla University, Hat Yai, Songkhla, 90110, Thailand; 3 Ministry of Health, Nay Pyi Taw, Myanmar

**Keywords:** Equity, Reproductive health, Youth, Accessibility, Utilization, Unmet need

## Abstract

**Background:**

Inequity of accessibility to and utilization of reproductive health (RH) services among youths is a global concern, especially in resource-limited areas. The level of inequity also varies by cultural and socio-economic contexts. To tailor RH services to the needs of youths, relevant solutions are required. This study aimed to assess baseline information on access to and utilization of RH services and unmet needs among youths living in resource-limited, suburban communities of Mandalay City, Myanmar.

**Methods:**

A community-based, cross-sectional study was conducted in all resource-limited, suburban communities of Mandalay City, Myanmar. A total of 444 randomly selected youths aged between 15 and 24 years were interviewed for three main outcomes, namely accessibility to and utilization of RH services and youth's unmet needs for these services. Factors associated with these outcomes were determined using multivariate logistic regression.

**Results:**

Although geographical accessibility was high (79.3%), financial accessibility was low (19.1%) resulting in a low overall accessibility (34.5%) to RH services. Two-thirds of youths used some kind of RH services at least once in the past. Levels of unmet needs for sexual RH information, family planning, maternal care and HIV testing were 62.6%, 31.9%, 38.7% and 56.2%, respectively. Youths living in the south or south-western suburbs, having a deceased parent, never being married or never exposed to mass media were less likely to access RH services. Being a young adult, current student, working as a waste recycler, having ever experienced a sexual relationship, ever being married, ever exposed to mass media, having a high knowledge of RH services and providers or a high level of accessibility to RH services significantly increased the likelihood of utilization of those services. In addition to youths’ socio-demographic characteristics, exposure to mass media, norm of peer exposure and knowledge on types of providers and services significantly influenced the unmet needs of youths towards RH services.

**Conclusion:**

Despite the availability of RH services, youth’s accessibility to and utilization of those services were unsatisfactory. The levels of youths’ unmet RH needs were alarmingly high.

## Background

Youths, defined as individuals aged between 15 to 24 years, constitute 18% of the world’s population, of which nearly 80% live in developing countries [1]. All youths have their own rights to access available reproductive health (RH) services and achieve a healthy reproductive life [2], which is also a keystone to achieving the Millennium Development Goals (MDG target 5b). Although RH services are important to youths, the accessibility to and utilization of RH services among youths are very limited due to various socio-economic and cultural disparities [3-5].

Low accessibility to and utilization of RH services creates a universal concern since unintended pregnancies, unsafe abortions, and sexually transmitted infections (STIs) have been shown to contribute to high morbidity and mortality rates, especially in developing countries [6-10]. Globally, approximately 16 million adolescents become pregnant every year, of which three million undergo unsafe abortions [11]. Adolescents are more likely to die from the causes related to pregnancy and childbirth compared to reproductive-aged women [11-13]. Likewise, the stigmatization of premarital sexual relations among young women deters them from seeking information about RH, engaging in safer sex and fulfilling their RH needs [14]. Those disparities of access to RH care affect not only the individuals but also their families, society and health systems as a whole at both national and global levels [15,16]. Therefore, the inequity of access to RH services between the rich and the poor, and those living in urban and rural areas are a global equity issue of high priority.

Although the availability of RH services has been promoted globally, the disparities of access to RH care still remain among youths, especially those living in resource-limited areas [17,18]. In addition, youth's accessibility to and utilization of RH services and their unmet needs for these services are influenced by various cultural and socio-economic contexts [19]. This study aimed to assess the level of accessibility to, utilization of and unmet needs for RH services and to identify the associated factors with respect to individual, family and various socio-economic characteristics among youths in resource-limited communities in suburban areas of Mandalay City, Myanmar.

## Methods

### Study area

Myanmar is located in South-East Asia, bordered by India, Bangladesh, China, Thailand and Laos. It is listed by WHO as a low-income country with an average GDP of $821. Of the 60 million residents, 32.7% have an income under the international poverty line [20]. Mandalay City, situated in central Myanmar, is a densely populated area with relatively low cultural, racial and social heterogeneity. There are 10 resource-limited communities situated in suburban areas of the city which are enriched with hard-to-reach, informal settings and residents with a low socio-economic status. All areas of Mandalay City are covered by RH projects and the availability of RH services has been ascertained; however, it is not known whether youths living in these areas have sufficient access to those RH services. Likewise, understanding their exact RH needs in order to tailor services to those needs remains a challenge for policy makers since there has been no previous study focusing on this vulnerable group.

### Study design and participants

A community-based, cross-sectional study was conducted from May 2011 to September 2011. Male and female youths aged between 15–24 years residing in any of the 10 resource-limited, suburban communities of Mandalay City were included in the study. Those who were not available for interviews or unwilling to participate in the study were excluded. The sample size was calculated based on an estimated utilization of RH services of 50% and adjusted for a 10% rate of incomplete information. Because of perceived variation of utilization levels among the different communities and between males and females, a design effect of 2 was also employed. Thus at least 215 female and 215 male youths, or a total of 430 youths, were required to estimate the utilization level with an accuracy of 10%.

### Data collection

#### Preparatory phase

The focus of the study was to estimate accessibility, utilization and unmet needs for RH services among youths. The study participants were interviewed using a structured questionnaire which was separated into five sections: socio-demographic characteristics and four main aspects of RH services (sexual RH (SRH) information, family planning, maternal care, STI/HIV testing). The questionnaire was developed based on several literature reviews on measuring accessibility [21-26], and the 2007 Indonesia Demographic and Health Survey (IDHS) which was undertaken as part of the international Demographic and Health Surveys project. The questionnaire from the IDHS is designed to collect data on fertility, family planning and maternal and child health [27].

The questionnaire was developed in English and back-translated from English to Burmese and English again by two independent translators. The meanings of all items in the questionnaire were checked accordingly and face validity was assessed. The interpretability and understanding of all items by the study participants were evaluated by pre-testing the questionnaire on 20 youths living in the study area but who were not included in the main study and the items were modified qualitatively. Six interviewers (3 females and 3 males) were trained on using a rapport approach and on how to conduct interviews. The interview process was standardized among all interviewers before data collection commenced.

### Sampling methods

Youths, in accordance with the inclusion and exclusion criteria in each community, were selected by simple random sampling. The process of sampling was conducted using the following steps. Firstly, all 10 resource-limited communities in all townships of Mandalay City were identified and confirmed by the township medical officers (TMOs). Secondly, lists of youths living in these communities were obtained from the community leaders. Thirdly, in each community, a roughly equal number of males and females were randomly selected using a random number generator based on the sex-stratified lists of youths living in each community. Finally, the appointments between the selected youths and the interviewers were made with the facilitation of community leaders.

### Data collection phase

The interviewers went to the youths’ houses at the scheduled appointment dates and times and conducted the interviews. For those who were not available at the first appointment, a second appointment was made. Before the interviews, all youths were informed about the objectives of the study and a signed consent form was obtained from each one. Privacy, anonymity and confidentiality were maintained throughout the process of this study. Discussions among the interviewers were made on a daily basis to check the accuracy of data.

### Outcome measures

The outcome measures were overall accessibility to RH services, overall utilization of RH services and youth's unmet needs for the four RH components mentioned previously.

### Accessibility to RH services

The accessibility in our study applied both geographical and financial considerations based on the youths’ own perception. Youths who lived within a 30-minute walk and within a one-mile distance from the nearest RH service centre from their home, and were aware that the service existed, were classified as having high geographical accessibility. For financial accessibility, we considered three aspects: travelling costs, service costs (drug fees, consultant fees) and youth’s perception of affordability. High financial accessibility was defined as a cost of travel less than 500 kyat ($0.63) and a cost of service less than 1500 kyat (US$1.9) with a perceived affordability of all costs. Youths having both high geographical and high financial accessibility were classified as having high overall accessibility and low accessibility otherwise.

### Utilization of RH services

Four essential components of RH services were assessed, namely SRH information services (any health education sessions/talks/classes that emphasized on SRH given in schools, clinics, or other places involving peer-based education excluding mass media), family planning services, comprehensive maternal care services (at least one antenatal care (ANC) visit, delivery by skilled birth attendance (SBA) and at least one postnatal care visit) and STI/HIV testing services.

Overall utilization of RH services was classified as the youths having sought or received at least one of the above four RH services. For SRH information and family planning, utilization must have been within the past 6 months for the youth to be classified as having utilized the service. For maternal health services and STI/HIV testing, if the youth used these services anytime in their life, they were classified as having utilized that service.

### Unmet RH needs

#### Unmet need for SRH information

All four RH components were evaluated for individual unmet needs. Since all youths have a right to access and utilize all SRH information services at any time in their life, unmet need for SRH information was defined as any youth who never utilized any SRH information service.

#### Unmet need for family planning

The standard definition given by WHO of unmet need for family planning applies to married and reproductive aged women only [28]. However, nowadays the need for family planning is discussed among women and their partners who are not ready to conceive, regardless of their marital status. As a result, unmet need for family planning in this study was defined as any currently non-pregnant, sexually active female youth (defined as having a current sexual partner and having sex with that partner) who did not use contraception, even though she did not intend to conceive. Intention to conceive was determined by asking the following question. *“Do you have a sexual partner?”* Those responding in the affirmative were further asked*, “Are you currently having sex with that partner?”* Those responding in the affirmative were further asked the following two questions: *“Do you use any type of contraception?”* and “*Do you want to conceive in the near future?”* Youths who responded “no” to the last question were classified as not intending to conceive.

#### Unmet need for maternal care

Any female youth who had been pregnant at least once and had never received comprehensive maternal care (defined as receiving all three components of maternal care) was defined as having an unmet need for maternal care.

#### Unmet need for STI/HIV testing

In this study, unmet need for STI/HIV testing was defined as any youth who had ever had sex without using a condom and had never been tested for STI/HIV. In addition, youths who did not use any RH service were asked, using an open-ended question, for their reason. The reasons were presented descriptively after coding and summarizing.

### Independent variables

Independent variables included demographic and socioeconomic characteristics, reproductive characteristics and family characteristics. Demographic and socio-economic characteristics included age, gender, ethnicity, religion, place of residence, schooling status, level of education, occupation, personal income, knowledge of RH services and types of providers, perceived norm of peer exposure to RH services, and exposure to any mass media on SRH via resources such as books, television, radio, or the Internet (never versus ever in their life). Youths were classified as either adolescent (age 15–19) or young adult (age 20–25). Education level was classified as none, low (less than secondary), middle (secondary) or high (more than secondary). Occupations were classified into 3 categories: unemployed (dependent/housewife/jobless), waste recycler (people who wander and collect used material all over the city and sell them to the recycling factories) and other employed (shopkeeper/municipal worker/labourer). Because waste cyclers were so common, their occupation was singled out. The international poverty line of $37.5 or 30,000 kyat (800 kyat per $1) was used as the threshold for a low personal income. Knowing the available types of services and types of providers within the area was regarded as knowledge of RH services and providers. Knowledge score was calculated by asking each youth if they knew of the available types of RH services and providers in their local area, which included a total of five types of services and eight types of providers. For each known type of service or provider, a youth was given one point reflecting a maximal score of 13. If the youths responded that his/her friends or relatives utilized any type of RH services, the perceived norm of peer exposure to RH services was classified as “yes”. Reproductive characteristics included past sexual experience, marital status, and pregnancy status. Family characteristics included parental status (alive or dead) and educational level, household income and number of family members.

### Statistical analysis

The percentages of accessibility to RH services, utilization of RH services, and unmet needs for the four SRH components were presented descriptively. The association between independent variables and the three main outcome measures was initially assessed by univariate analysis. Independent variables which had a *p*-value of less than 0.2 were included in the multivariate logistic regression analysis. Statistical significance was considered if a variable had a *p-*value less than 0.05 in the final model. All analysis was done using R.

### Ethical considerations

The proposal was approved by the Institutional Ethics Committee of the Faculty of Medicine, Prince of Songkla University, Hat Yai, Thailand and the Ethics Committee of the Department of Medical Research (Upper Myanmar) before the study was conducted. All eligible youths were informed of their right to participate in the study using informed consent and their anonymity and confidentiality was stringently maintained throughout the study.

## Results

### Socio-demographic characteristics

All sampled youths agreed to participate in the study and completed the interviews for a response rate of 100%. The demographic, socio-economic, reproductive and family characteristics of the youths are shown in Table 1. Of 444 youths interviewed, 215 were males and 229 were females. Among them, 231 (52%) were adolescents and 213 (48%) were young adults. Almost all were Buddhists and of Bamar ethnicity. Most (88%) were out of school, and very few attained a high-school level of education. Unemployment rate was considerably high (21%). One-fourth worked as waste recyclers. The personal income of the youths was very low and nearly half had an income under the international poverty line. Although there was a high prevalence of positive perceived norm of peer exposure to RH services, the youths had very little knowledge of available types of provider and RH services in their communities. Nearly one-third had never accessed any type of mass media (books/magazines, TV, radio or the Internet) for information on RH. More than half (53%) had a history of sexual exposure and less than half (44%) had ever been married. The majority (70%) of the youths, or their spouses, had never been pregnant. One-fourth of the youths had one deceased parent (mostly their fathers, who passed away before age fifty). Parental education level was mostly low. The average household income was US$ 178. 

**Table 1:** Characteristics of youths

**Factor**	**n (%)**	**Factor**	**n (%)**
**Demographic and socioeconomic characteristics**		**Reproductive characteristics**	
Age group		Sexual exposure	
Adolescents	231 (52.0)	Never exposed	209 (47.1)
Young adults	213 (48.0)	Ever exposed	235 (52.9)
Sex		Marital status	
Male	215 (48.4)	Never married	250 (56.3)
Female	229 (51.6)	Ever married	194 (43.7)
Ethnic group		Pregnancy status*	
Bamar	437 (98.4)	Never pregnant	313 (70.5)
Other	7 (1.6)	Ever pregnant	109 (24.5)
Religion		Currently pregnant	22 (5.0)
Buddhist	438 (98.6)		
Other	6 (1.4)	**Knowledge, perception and media exposure**	
Place of residence		Perceived norm of peer exposure	
East	93 (20.9)	No	55 (12.4)
North-west	50 (11.3)	Yes	389 (87.6)
West	99 (22.3)	Knowledge of RH services and providers	
South-west	40 (9)	Mean (SD)	3.3 (1.6)
South	128 (28.8)	Media exposure	
South-east	34 (7.7)	Ever exposed	302 (68.0)
Schooling status		Never exposed	142 (32.0)
In-school	54 (12.2)		
Out-of-school	390 (87.8)	**Family characteristics**	
Education		Parental status	
None	25 (5.6)	Both alive	281 (63.2)
Low	192 (43.2)	One or more deceased	163 (36.8)
Middle	128 (28.8)	Father's education level	
High	99 (22.3)	None	96 (21.6)
Occupation		Low	259 (58.3)
Unemployed	92 (20.7)	Middle and above	89 (20.1)
Waste recycler	103 (23.2)	Mother's education level	
Other employed	249 (56.1)	None	93 (20.9)
Personal income (US$)		Low	320 (72.1)
≤37.5	175 (39.4)	Middle and above	31 (7.0)
>37.5	269 (60.6)	Household income (US$)	
		Mean (SD)	178 (96.1)
		Number of family members	
		≤5 persons	246 (55.4)
		>5 persons	198 (44.6)

* For males, responses are for their spouses or partners.

### Accessibility to RH services

The majority of the participants said that there was at least one RH service centre within a 30-minute walk (94%) and within one mile (80%) from their home. The most common mode of transportation to the RH centre was by foot (83%). Only 5% said that the service was free of charge, 20% said that they had to pay less than $1.29, while 75% said that they had to pay more than $1.29. Based on the youth’s self perception, 69% thought that all costs (travelling and service) were not affordable.

A summary of the geographical, financial and overall accessibility and utilization levels for males and females are presented in Table 2. Most had a high level of geographical accessibility (79%) and a low level of financial accessibility (81%). Most had a low overall level of accessibility (66%). There was no difference between males and females in all three aspects of accessibility. 

**Table 2:** Comparison of accessibility and utilization between male and female youths

	**Male, n (%)**	**Female, n (%)**	**Total, n (%)**
**Geographical accessibility**			
Low	47 (21.9)	45 (19.7)	92 (20.7)
High	168 (78.1)	184 (80.3)	352 (79.3)
**Financial accessibility**			
Low	170 (79.1)	189 (82.5)	359 (80.9)
High	45 (20.9)	40 (17.5)	85 (19.1)
**Overall accessibility**			
Low	140 (65.1)	151 (65.9)	291 (65.5)
High	75 (34.9)	78 (34.1)	153 (34.5)
**Overall utilization**			
No	84 (39.1)	62 (27.1)	146 (32.9)
Yes	131 (60.9)	167 (72.9)	298 (67.1)

Table 3 shows the final model of factors associated with overall accessibility. Youths living in the south (adjusted OR (AOR) 0.29, 95% confidence interval (CI) 0.16-0.52) or south-western (AOR 0.36, 95% CI 0.15-0.84) suburbs, having one or more deceased parent (AOR 0.5, 95% CI 0.31-0.81), never being married (AOR 0.57, 95% CI 0.36-0.90) or having never been exposed to mass media (AOR 0.55, 95% CI 0.35-0.86) were less likely to have a high overall accessibility to RH services. 

**Table 3:** Factors significantly associated with overall accessibility to RH services

**Factor**	**Adjusted OR (95% CI)**	P-value*
**Youth's place of residence:** ref.= East		< 0.001
North-west	0.52 (0.25-1.08)	
West	1.05 (0.60-1.83)	
South-west	0.36 (0.15-0.84)	
South	0.29 (0.16-0.52)	
**Parental status:** ref.= Both alive		0.004
One or both deceased	0.50 (0.31-0.81)	
**Youth's marital status:** ref.= Ever married		0.015
Never married	0.57 (0.36-0.90)	
**Youth’s media exposure:** ref.= Ever exposed		0.01
Never exposed	0.55 (0.35-0.86)	

Factors included in the initial model were age group, personal income, father’s education, mother’s education and history of sexual exposure.

OR: Odds Ratio; CI: Confidence Interval; * Likelihood ratio test.

### Utilization of RH services

Of the 444 youths, 67% had ever utilized at least one type of RH service. The most utilized service was family planning (70%). Common service providers were midwives (39%) and non-governmental organizations (NGOs) (22%). Table 4 depicts the factors associated with the overall utilization of any RH services. Being a young adult, current student, working as a waste recycler, having a history of sexual exposure, ever being married, exposure to mass media, having a high knowledge of RH services and providers or having a high level of accessibility to RH services significantly increased the likelihood of utilizing those services. The difference in utilization between males and females was statistically significant (Table 2). 

**Table 4:** Factors significantly associated with utilization of at least one RH service

**Factor**	**Adjusted OR (95% CI)**	P-value*
**Age group:** ref = Adolescents		0.039
Young adults	1.82 (1.03-3.22)	
**Schooling status:** ref = Out-of-school		< 0.001
In-school	5.88 (2.44-14.28)	
**Occupation:** ref = Unemployed		0.003
Waste recycler	4.00 (1.67-9.58)	
Employed	1.68 (0.82-3.45)	
**Sexual exposure:** ref = Never exposed		0.006
Ever exposed	3.12 (1.35-7.24)	
**Marital status:** ref = Never married		0.004
Ever married	3.72 (1.55-8.91)	
**Media exposure:** ref = Never exposed		0.006
Ever exposed	2.20 (1.25-3.86)	
**Knowledge of RH services and providers**	1.43 (1.18-1.73)	< 0.001
**Overall accessibility:** ref = Low		0.013
High	1.96 (1.14-3.36)	

Factors included in the initial model were sex, place of residence, education attainment, personal income, parental status, father’s education, mother’s education, pregnancy status and perceived norm on peer exposure.

OR: Odds Ratio; CI: Confidence Interval; * Likelihood ratio test.

### Unmet RH needs

#### Unmet need for SRH information

The level of unmet need for SRH information was 67%. Of all youths, 33% had accessed or received SRH information in their lifetime, of which NGOs were the most common providers (67%). Youths who had attended RH education sessions said that the most common types of information provided were for STI/HIV (90%) and contraceptives (46%). The main reasons of non-exposure to SRH information were the youth’s own feeling of embarrassment and negative attitudes of the youth's guardians.

#### Unmet need for family planning services

Of 113 sexually active female youths who were currently neither pregnant nor intending to be pregnant, 36 were not using any form of contraceptive, giving a level of unmet need for family planning services of 32%. Of the 77 who were currently using contraceptives, the most common types of contraceptives used were depot injections (51%) and oral contraceptive pill (26%). Family planning service centres utilized by the youths included private clinics (21%), midwives (17%), drug stores (13%) and NGOs (11%). Common reasons for not utilizing family planning services were an inability to pay for the services and confidentiality concerns of unmarried youths. Less common reasons included inconvenience of transportation, as well as negative attitudes of providers.

#### Unmet need for maternal care services

The rate of unmet need for comprehensive maternal care was 39%. Out of 75 female youths who had ever been pregnant, 13% had never received antenatal care (ANC), 17% had deliveries that were never assisted by an SBA and 25% had never received postnatal care. Common reasons for not utilizing comprehensive maternal care services were inability to pay for the service and inconvenience of transportation.

#### Unmet need for STI/HIV testing

Among 119 youths who had had unprotected sexual encounters, the level of unmet need for STI/HIV testing was 50%. Of those who had been tested, the usual places were urban health centres (32%), the STI clinic of Mandalay General Hospital (29%) and NGOs (26%). Common reasons for having STI/HIV tests were pregnancy (54%), curiosity (14%) and having practised a risky sexual behaviour (14%). The most common reason for not having an STI/HIV test was lack of perceived need to do so. Less common reasons were fear of getting a positive test result, embarrassment, not knowing the location of the testing facility, and inability to pay for the service.

### Factors associated with unmet needs for RH services

Table 5 shows factors associated with unmet needs for all four components of RH services. A lower unmet need for SRH information was found among young adults (AOR 0.55, 95% CI 0.34-0.91), youths who had a high accessibility to RH services (AOR 0.61, 95% CI 0.39-0.94) and a high knowledge of RH services and providers (AOR 0.78, 95% CI 0.68-0.90), while out-of-school youths were more likely to have an unmet need for SRH information (AOR 4.47, 95% CI 2.30-8.68). 

**Table 5:** Factors significantly associated with unmet needs for RH services

**Factor**	**SRH information**	**Family planning**	**Maternal care**	**HIV/STD testing**
	**OR (95% CI)**	**OR (95% CI)**	**OR (95% CI)**	**OR (95% CI)**
**Age group:** ref. = Adolescents		-	-	
Young adults	0.55 (0.34 – 0.91)†			0.43 (0.21 – 0.86)†
**Youth's residence:** ref. = East	-	-	-	
North-west				1.40 (0.49 – 4.01)
West				0.75 (0.33 – 1.7)
South- west				5.14 (1.68 – 15.75)‡
South				3.16 (1.40 – 7.16) ‡
**Schooling status:** ref. = In-school		-	-	-
Out-of-school	4.47 (2.30 – 8.68)*			
**Marital status:** ref = Never married	-		-	-
Ever married		0.09 (0.01 – 0.97)*		
**Number of family members:** ref = ≤5 persons	-	-		-
>5 persons			2.6 (1.25 – 5.37)‡	
**Overall accessibility:** ref = Low		-	-	-
High	0.61 (0.39 – 0.94)†			
**Media exposure:** ref = Never exposed	-		-	-
Ever exposed		0.21 (0.05 – 0.83)†		
**Knowledge on RH services**	0.78 (0.68 – 0.90)*	0.61 (0.41 – 0.89) ‡	-	0.72 (0.59 – 0.88)*
**Perceived norm of peer exposure to RH services:** ref = No	-	-		
Yes			0.09 (0.01 – 0.76)‡	0.16 (0.05 – 0.54)*

Notes: † p ≤ 0.05; ‡p ≤ 0.01; *p ≤ 0.001 (Likelihood ratio test) OR: Odds Ratio; CI: Confidence Interval.

Factors included in each initial model were sex, education attainment, occupation, personal income, parental status, father’s education, mother’s education, household income, history of sexual exposure, pregnancy status.

Preventative factors significantly associated with unmet need for family planning were being married (AOR 0.09, 95% CI 0.01-0.97), exposure to mass media (AOR 0.21, 95% CI 0.05-0.83) and having a high knowledge of RH services and providers (AOR 0.61, 95% CI 0.41-0.89).

Youths who lived in a family having more than 5 family members had a higher likelihood of having an unmet need for maternal health services (AOR 2.60, 95% CI 1.25-5.37) while those who had a perceived norm of peer exposure to RH services had a lower likelihood (AOR 0.09, 95% CI 0.01-0.76).

Youths who lived in the south (AOR 3.16, 95% CI 1.40-7.16) or south-western (AOR 5.14, 95% CI 1.68-15.75) suburbs had a higher unmet need for STI/HIV testing services, whereas preventive factors were being a young adult (AOR 0.43, 95% CI 0.21-0.86), having a perceived norm of peer exposure to RH services (AOR 0.16, 95% CI 0.05-0.54) and having a high knowledge of RH services and providers (AOR 0.72, 95% CI 0.59-0.88).

## Discussion

Although the level of accessibility to reproductive health services by geographical assessment among youths in our study was high, the financial accessibility was low reflecting a low overall level of utilization of all RH services. Unmet needs for RH services were consistently high for all four aspects. In addition to youth’s individual and socio-economic characteristics, some important factors such as exposure to mass media, knowledge of RH services and providers and perceived norm of peer exposure to RH services, significantly influenced the youth’s accessibility to and utilization of RH services and their unmet needs for these services.

A low level of accessibility to RH services due to financial aspects was found in our study although the geographical accessibility was adequate. Geographic and financial assessments are commonly used for measuring accessibility to health care services; however, the methods of measurement vary [29,30]. Accessibility to health care is influenced not only by geographical but also by social, psychological, economic and organizational factors [22,23]. Geographical Information Systems (GIS) have been commonly used to measure physical accessibility [10,31]; however, financial considerations and client’s perceptions of their own access have been lacking. Even if geographical accessibility of health services is adequate, the youths won’t access them unless they know that they exist. Other studies have suggested that the perception of a client’s own geographical and financial access to health care is also important regardless of actual time or distance involved in reaching the service [22-25]. Therefore, accessibility in our study applied both geographical and financial considerations based on the youths’ own perceptions. Methods used to measure accessibility in our study were quite similar to those from the studies conducted in Thailand, Tanzania and Nepal [22,23,26].

We found that geographical accessibility was satisfactory in our study compared to other studies [21-23,26]. To date, little information is available on issues of accessibility to RH services in Myanmar. However, an RH report from the Ministry of Health, Myanmar, which surveyed three townships during 1991 to 2001, mentioned that the distance and travelling time were not the main barriers of access to RH services whereas financial impoverishment hindered utilization of contraceptives among the poor [32]. These findings indicate that inaccessibility to RH services due to the financial aspects has been present for a decade. In contrast to our study, distance and travelling time from home to RH services remain important barriers in rural areas of Nepal, the Philippines and Malawi [26,33,34]. Low accessibility to RH services among the poor youths due to a lack of affordability in our study highlighted the inequity of access to RH services between the rich and the poor, and the rural and urban dwellers as found in previous studies [8,35,36]. In addition, our study confirmed the finding from other studies that low accessibility to RH services results in low utilization of those services [25,26,37,38].

The measurement of utilization of RH services varies widely. Some studies used just one aspect of RH services [39-42] while others used multiple aspects [43-45]. However, very few studies assessed all four essential aspects of RH services as we did in our study. Even among few studies which considered multiple aspects of RH, the services assessed varied. In 3 studies conducted in the USA [43-45], the level of utilization was found to be similar to our study; however, the American studies included utilization of RH services that were not assessed in our study, such as PAP smear, pelvic examination and abortions services.

In our study, the level of unmet need for family planning was higher than that among reproductive-aged women in Myanmar and other South East Asian countries [46,47] but lower than that in Pakistan [48]. Inability to pay was the main reason for youths in our study to have a higher unmet need for family planning compared to women from other South East Asian countries. Religious beliefs deterred women in Pakistan from using contraceptives causing them to have a higher unmet need for family planning.

There is currently no standard definition of unmet needs for SRH information, comprehensive maternal care and STI/HIV testing service. No previous study has assessed the levels of unmet need for those services; therefore no comparison can be made. In our study, we calculated those unmet needs based on the actual needs of youths. A broad approach to defining actual needs for those RH services is warranted so that the unmet needs can be calculated in the future. However, youths’ perceived needs of those services also played a major role. In our study, we found that a majority of the youths wanted to receive SRH information and maternal care services even though they perceived that they could not afford them or were too embarrassed to use the service. In contrast, a majority of youths who had had sex without using any condoms to prevent STI/HIV lacked a perceived need to receive STI/HIV testing despite their actual need for it.

In general, the factors associated with accessibility to, utilization of, and unmet needs for RH services were interrelated. Youths living in the south and south-western suburbs of Mandalay City had a lower level of accessibility and a higher unmet need for HIV testing services. Possible explanations are that the communities in these areas are newly settled, and have poor road conditions, especially in the rainy season, compared to other areas. This fact was supported by the youth’s stated barriers for utilization of RH services, i.e. difficulties with transportation. A higher utilization of RH services among married youths or those who were in a sexual relationship might be due to them having good spousal communication [49] or perception of their susceptibility of RH problems as their perceived needs [50].

In our study, adolescents and out-of-school youths had a significantly lower utilization and higher unmet needs for SRH information. SRH information is usually obtained in the regular school-based SRH education classes in Mandalay City, thus out-of-school youths have fewer opportunities to access this information [51]. In addition, adolescents’ own embarrassment and negative attitudes of their guardians have been shown to be common barriers to access SRH information services [14,19,52], findings which were similar to ours.

Youths who had never been married had a significantly lower utilization and higher unmet need for family planning. A concern about confidentiality by unmarried youths was the main barrier to access the service in our study as well as in many other studies [53-55]. The perception on society’s and providers’ negative attitudes on receiving the service is also an important barrier [55,56]. Fulfilling the need for family planning services among unmarried youths is critical since the rate of unwanted pregnancy and unsafe abortion among unmarried youths each year is alarmingly high [53,57]. A higher likelihood of RH utilization was found among youths working as waste recyclers compared to the unemployed in our study. One reason could be due to the active involvement of NGOs and government assigned midwives who provide RH services to the areas where this vulnerable group live.

In our study, we found that family factors, as well as individual and reproductive factors, played an important role in youths’ utilization of RH services. Youths who had one deceased parent and those who had more than five family members were less likely to utilize the services. This finding was supported by one study that found that the relationship between youths and their parents, as well as childhood family conditions, influenced the youth’s utilization of RH services [44]. Moreover, having a high number of family members in the household may increase the financial burden of the youth’s family.

The finding that a higher knowledge of available RH services by the youths increased their odds of utilizing those services was supported by a similar study in Ethiopia which measured youths’ knowledge of RH services and providers [58]. If the youths are more aware of the services and providers in their local area, they are more likely to use those services.

Perceived norm of peer exposure to RH services was significantly associated with lower unmet need for maternal care and STI/HIV testing, a finding similar to a study conducted in Nepal and China [19,59]. This result could be explained by the Theory of Reasoned Action [60], in which a person's attitude, combined with subjective norms, forms their behavioural intention. If a youth has a friend who uses maternal care services or STI/HIV testing, the youth is more likely to also use that service, either through direct encouragement, or the influence of social norms.

Media exposure was consistently associated with a higher odds of accessibility to, and utilization of RH services and a lower odds of having an unmet need for these services by the youths in our study. Media exposure was also shown to be a significant factor for access to SRH information among male youths in India [61], SRH information and contraceptives use in China [62] and contraceptives use in the Philippines [33]. In addition, the media for SRH information was shown to be the most common source for unmarried migrant youths in three major cities of China [63]. These results indicate that media such as books, radio, television and the Internet are important for promoting utilization of RH services among young people.

### Strengths and limitations

A holistic view of the accessibility to and utilization of RH services and youth’s unmet needs towards RH services was elicited in our study. The challenges and factors hindering access to RH services among youths living in hard-to-reach, resource-limited suburban communities were also highlighted. The questions used in the questionnaire were modified from the International Demographic Health Survey and we also used a multidimensional approach for accessibility to RH services, i.e. geographical and financial aspects. In addition, all outcome measures were based on either standard definitions or actual needs of the youths. The triangulation of factors associated with outcomes identified from regression analysis was consistent with youth’s own reasons. There were some limitations in our study. First, the three main outcomes were obtained from the youth’s own perceptions and responses, and were not objectively measured or confirmed by medical reports. However, anonymity and confidentiality were stressed and the youths were encouraged to answer all questions truthfully. Second, the results cannot be generalized to all youths living outside resource-limited communities because the demographic and socio-economic characteristics of youths living outside our study area may be different. Third, even though the community leaders, with the help of municipal authorities, provided a list of all youths living in their communities, it is possible that some youths may have not been included in the lists. Lastly, the level of unmet need for STI/HIV testing services was not assessed according to the youth’s risky sexual behaviours since sexual behaviour is a culturally sensitive issue in Myanmar.

## Conclusions

Despite the high availability of RH services, accessibility to and utilization of the services among youths in the study area were unsatisfactory. The levels of unmet need for RH services, especially family planning, were alarmingly high. Common associated factors of access to, utilization of and unmet need for RH services were place of residence, marital status, exposure to mass media, knowledge about the services and providers and perceived norm of peer exposure to RH services.

Financial constraint and affordability of youths to access and utilize RH services is an urgent issue for national policy makers and all stakeholders. Improvement of youth’s knowledge about the existence of RH services is essential. Recent RH services should be tailored to the special needs of adolescents and unmarried youths. Dissemination of SRH information via popular mass media, such as television, and formal and informal education programs to enhance utilization and decrease unmet needs in these resource-limited areas is strongly advised. Further studies should emphasize on the effectiveness of the mass media on enhancing utilization of those services among youths in these areas.

## Competing interests

The authors declare that there are no competing interests concerning this research article.

## Authors’ contributions

PPTZ designed the study, conducted the data collection process, analyzed and interpreted the data, and prepared the manuscript. TL provided supervision on all aspects of the study and manuscript preparation. TTH helped to conceptualize the study, supported the data collection process and commented on the manuscript. EM helped with data management, statistical analysis and manuscript preparation. All authors read and approved the final manuscript.

## Pre-publication history

The pre-publication history for this paper can be accessed here:

http://www.biomedcentral.com/1472-6963/12/458/prepub
